# Exploring the Continuum of Hypertrophic Cardiomyopathy—From DNA to Clinical Expression

**DOI:** 10.3390/medicina55060299

**Published:** 2019-06-23

**Authors:** Nicoleta Monica Popa-Fotea, Miruna Mihaela Micheu, Vlad Bataila, Alexandru Scafa-Udriste, Lucian Dorobantu, Alina Ioana Scarlatescu, Diana Zamfir, Monica Stoian, Sebastian Onciul, Maria Dorobantu

**Affiliations:** 1 Department of Cardiology, Clinical Emergency Hospital of Bucharest, Floreasca Street 8, 014461 Bucharest, Romania; fotea.nicoleta@yahoo.com (N.M.P.-F.); vladbataila@yahoo.co.uk (V.B.); alexscafa@yahoo.com (A.S.-U.); alina.scarlatescu@gmail.com (A.I.S.); diana_zam74@yahoo.com (D.Z.); monica.predescu@gmail.com (M.S.); sebastian.onciul@gmail.com (S.O.); maria.dorobantu@gmail.com (M.D.); 2 Department 4-Cardiothoracic Pathology, University of Medicine and Pharmacy Carol Davila, Eroii Sanitari Bvd. 8, 050474 Bucharest, Romania; 3 Cardiomyopathy Center, Monza Hospital, Tony Bulandra Street 27, 021968 Bucharest, Romania; ludorobantu@gmail.com

**Keywords:** hypertrophic cardiomyopathy, causative mutations, modifiers, cardiac imaging

## Abstract

The concepts underlying hypertrophic cardiomyopathy (HCM) pathogenesis have evolved greatly over the last 60 years since the pioneering work of the British pathologist Donald Teare, presenting the autopsy findings of “asymmetric hypertrophy of the heart in young adults”. Advances in human genome analysis and cardiac imaging techniques have enriched our understanding of the complex architecture of the malady and shaped the way we perceive the illness continuum. Presently, HCM is acknowledged as “a disease of the sarcomere”, where the relationship between genotype and phenotype is not straightforward but subject to various genetic and nongenetic influences. The focus of this review is to discuss key aspects related to molecular mechanisms and imaging aspects that have prompted genotype–phenotype correlations, which will hopefully empower patient-tailored health interventions.

## 1. Introduction

**Figure 1:**
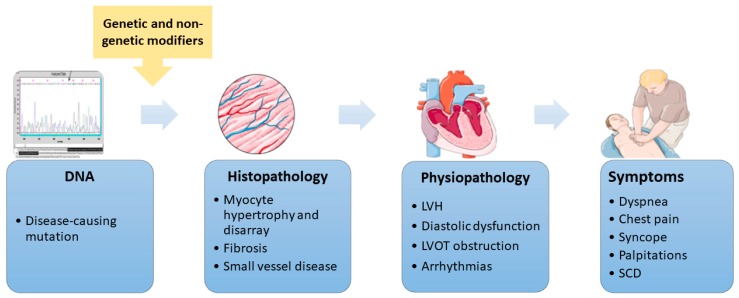
The hypertrophic cardiomyopathy continuum. The pathway from the disease-causing mutation to clinical expression is subject to various genetic and nongenetic influences. This figure was created with images from Servier Medical Art by Servier. DNA—deoxyribonucleic acid, LVH—left ventricular hypertrophy, LVOT—left ventricular outflow tract, and SCD—sudden cardiac death.

Hypertrophic cardiomyopathy (HCM) is an autosomal dominant disease caused by mutations in sarcomeric or sarcomeric-related genes [1]. It is the most common inherited cardiomyopathy, with a prevalence in the general population of 1:500, or even higher, as suggested by recent evidence [1,2]. There is also the possibility of spontaneous/de novo mutations, recognized and reported in literature since the early 1990s [3,4]. Notably, HCM is highly heterogeneous as concerns clinical expression and outcome, even within the same family. Clinical manifestations vary greatly, from asymptomatic to severe forms, or even sudden cardiac death (SCD), which may be the first expression of the disease. Incomplete penetrance and variable expressivity are the result of various genetic and nongenetic influences that are not yet fully understood (Figure 1).

The condition is defined by the presence of left ventricular hypertrophy (LVH) that is not solely explained by abnormal loading conditions [5]. Several other disorders (with different genetic etiology) are also characterized by LVH and its inherent aftermaths. Apart from sharing the key feature (i.e., LVH), these illnesses (denoted as HCM phenocopies) differ from HCM not only in terms of the genes involved but also in terms of natural history, patients’ management, and prognosis. In the following, we will refer only to sarcomeric HCM, leaving aside the above-mentioned phenocopies.

## 2. Etiology

### 2.1. Current Knowledge

In patients satisfying diagnostic criteria, genetic testing is recommended (Class I, level of evidence B) [6]. Mutations in genes encoding myosin binding protein C (*MYBPC3*) and *β*-myosin heavy chain (*MYH7*) cause almost 75% of the genotyped cases. Other genes involved in HCM less frequently encountered are: cardiac troponin T (*TNNT2*) and I (*TNNI3*), α-Tropomyosin (*TPM1*), α-Actin 1 (*ACTC1*), and myosin light chain (*MYHL3* and *MYHL2*). Ninety-nine percent of the pathogenic variants are found in the aforesaid genes outlined in literature as “core genes” [6,7] (Table 1).

Sustained efforts have been made to ascertain genotype–phenotype correlations, particularly in terms of prognosis. Early studies suggested that mutations in *MYH7* gene were generally associated with a worse prognosis compared to *MYBPC3* mutations; also, they indicated the existence of “malignant” variants causing more severe phenotypes and reduced survival, as opposed to “benign” ones that were associated with normal life expectancy [27,28]. Later work in large cohorts challenged the notion of locus-specific clinical outcomes [8,29]. Present evidence advocates that the HCM phenotype is independent of underlying gene mutation, with no clear connection between genotype and disease expression as regards the extent of LVH and occurrence of SCD.

In the vast majority of cases, the malady is caused by a single heterozygous mutation, and rarely (3% to 5%) have multiple mutations been detected (either on both copies of the gene or in different genes), resulting in particularly severe hypertrophy, a younger age of onset (often <10 years), and more adverse events (i.e., SCD) [30,31,32,33,34,35]

Apart from the main genes mentioned, there are also emerging genes found in a minority of HCM cases, whose definite disease causality is yet to be determined [19,20,36].

### 2.2. Challenges of Mutation Analysis

The use of high-throughput sequencing techniques together with extensive gene panels in daily practice have greatly contributed to discovery of new causal mutations, but they have also increased the chances of spotting variants of uncertain significance (VUS), which are difficult to interpret. Discriminating between a pathogenic variant and a rare variant with no clinical significance is challenging, especially in the case of “private” mutations, which are unique for a family. Classification of the new identified variants has to be done with extreme caution since the result of genetic testing directs the management of first-degree relatives.

Also, in particular cases, the accurate location of the mutation is not an easy task. Indeed, our group recently reported the challenges faced in mapping a deletion in the *MYBPC3* gene identified in an HCM patient. The mutation (p.Lys814del) was initially detected in a next-generation sequencing (NGS) screening, and it was subsequently confirmed by Sanger sequencing (GenBank accession number MH595891) [37]. Because of the particular nucleotide structure of the affected sequence comprising a short tandem repeat of four Lys residues, in spite of using different bioinformatics tools, it was not possible to precisely determine which triplet was in fact deleted. One should be aware of these findings when reporting the genomic coordinates of such a type of deletion.

## 3. Histopathological Hallmarks

While septal hypertrophy was initially described and is still the most common localization [38], today it is widely acknowledged that wall thickening can involve any area of the myocardium. In these areas, a wide array of tissue histopathological abnormalities are present. The morphological centerpieces of HCM are myocyte hypertrophy and disarray [39], myocardial fibrosis, extracellular matrix abnormalities [40], and small vessel disease [41].

Affected cardiac muscle cells present with shape modification, sometimes severe, and also with an increase in size mainly by their transverse diameter [42,43], but they maintain their intercellular connections [42]. The cardiac myocytes form an abnormal tissue architecture, resulting in severe disarray. They have a disorganized pattern with oblique, perpendicular, herringbone, or sometimes as severe as completely irregular. Bel-Kahn J. showed in an initial study that cardiac myocyte disarray is also present in various other heart diseases with structural implications but with a more reduced degree of disarray [44]. In an inspiring study, Varnava et al. showed that diseased heart tissue consisted of 10% to 90% cell disarray, which did not correlate with total heart weight [45]. The affected area was located adjacent to perfectly normal tissue architecture. The patchy nature of normal and diseased tissue was present in all patients from the study. Also, the myocyte disorder was seen in a greater proportion in patients without mitral valve systolic anterior motion (SAM) and was inversely correlated with fibrosis; therefore, it had a much higher proportion in young patients [45,46]. Another important consequence of cardiac muscle cell disorder is intercellular junction abnormalities. Sepp et al. showed that desmosomes in the affected areas had pronounced derangements. Because of the severely distorted anatomy, some of them merged and formed “huge megadiscs” but keeping side-to-side connections. The gap junctions also showed abnormalities in affected areas, as these were found in a diffuse pattern over the surface of the myocyte, not just in intercalated disks [47]. Although not proven in vivo, intercellular junction abnormalities could cause, or certainly contribute, to diastolic dysfunction and, most importantly, to the infamous ventricular arrhythmias.

Another morphological key piece of HCM is the small vessel disease that sometimes accompanies the myocardial cell disarray. Maron et al. describes in a cornerstone study that intramural arteries are characterized by wall thickening due to proliferation of intima and medial components via increased collagen formation and smooth muscle cell activity. Altered arteries were present in 83% in HCM patients compared to 9% of controls. The arteries had a significantly larger external diameter, with a higher grade of thickening and narrower lumen, found more frequently in areas with fibrosis. Also, there is a high degree of possibility for small vessel disease to be present, along with myocardial disarray, from childbirth [41,46]. This microvascular disease is probably responsible for myocardial ischemic aggression with patchy myocardial infarctions, myocyte death, and repair.

Myocardial fibrosis is the last histopathological hallmark of HCM. It is common both on a microscopic and a macroscopic level. It is a logical consequence of the imbalance between the increased demand of the hypertrophied myocardium and the poor supply offered by the diseased intramural blood vessels. Myocardial scaring is one of the main factors leading to impaired diastolic function and premature death [48]. Myocardial fibrosis is significantly more common in HCM patients; Shirani et al. demonstrated that in children and young adults with HCM, who were asymptomatic before sudden cardiac death, there was three to eight times more fibrous tissue at the level of the ventricular septum than in hypertensive and healthy subjects, respectively [49]. On the other hand, defying the classical theory of ischemia being the main cause of fibrosis, Ho et al. demonstrated that in HCM patients, fibrosis is a separate process that evolves simultaneously to myocyte disarray and microvascular disease, before overt hypertrophy is present. Mutation gene carriers without LVH and without fibrosis detected through late gadolinium enhancement (LGE) displayed significantly more fibrous tissue compared with normal patients, but less than those with overt disease. By comparing the serum levels of C-terminal propeptide of type I collagen (CICP) to the collagen type I C-terminal telopeptide (ICTP), Ho concluded that collagen synthesis exceeded degradation only in patients with overt disease [40]. Another hypothesis for myocardial fibrosis development is that numerous stress factors, like mechanical stress due to disorganized sarcomeric and cellular architecture, myocardial ischemia, or neuroendocrine activation, lead to an increase in nuclear factor kappa—a transcription factor regulating inflammation. That in turn gives rise to inflammatory cell activation, including fibroblasts, finally leading to myocardial fibrosis [50].

Starting from the primary gene mutation, followed by impaired protein expression and defective sarcomere assembly, and arriving to post-translational modifications and mitotic factors [51], histopathological elements are the key transition points that link genotype with phenotype and clinical expression.

## 4. Clinical Expression

The morpho-pathological changes in HCM determine various hemodynamic consequences, affecting the efficacy of normal heart function. HCM patients can display one or more hemodynamic abnormalities, depending on the stage of the disease.

Impaired LV filling and diastolic dysfunction occur secondary to decreased chamber compliance, heterogeneous ventricular relaxation, and loss of normal ventricular suction. The left atrium (LA) is also enlarged [52]. The systolic function may be also modified with a high-normal or elevated left ventricular ejection fraction (LVEF) due to increased contractility and reduced end-systolic volume. The hemodynamics of the right ventricle are rarely affected.

Dynamic left ventricular outflow tract (LVOT) obstruction is caused by LVH, abnormal subvalvular mitral disposition, and a modified ventricular configuration. The LVH is frequently asymmetrical, and other patterns (concentric, apical, lateral wall) are less common. Systolic anterior motion of the anterior leaflet of the mitral valve occurs when the anterior or both leaflets of the mitral valve are absorbed into the LVOT [53]. Theoretically, LVOT obstruction is considered significant if a gradient >30 mmHg is recorded [54]. It is estimated that one-third of patients with HCM have LVOT obstruction at rest [55].

HCM patients can experience myocardial ischemia caused by structural alterations of the intramural coronary arteries [41].

Changes in the heart’s hemodynamics are the substrate of clinical presentation, which is highly variable—patients are often asymptomatic or mildly symptomatic. When present, the most common clinical manifestations include dyspnea, angina, syncope, palpitations, and even SCD, each one of these being the expression of various pathophysiologic conditions [1,56].

Dyspnea, varying from dyspnea on exertion to nocturnal paroxistic dyspnea or even orthopnea, is the clinical expression of heart failure (usually with preserved LVEF) and the most common symptom [6]. Dyspnea can result from a variety of mechanisms: diastolic dysfunction due to myocardial hypertrophy, elevated LV filling pressures, outflow tract obstruction, myocardial ischemia, mitral regurgitation, and, last but not least, LV systolic dysfunction that might occur as the disease progresses [1,56,57].

HCM patients often experience chest pain at rest or on exertion, usually the expression of microvascular dysfunction [51]. One can observe, on one hand, a reduction of myocardial blood supply determined by the compression of intramural vasculature or by LVOT obstruction and, on the other hand, an increased oxygen demand of the hypertrophied myocardium leading to symptoms suggestive of angina (typical or atypical) [6,51].

Syncope, another possible clinical manifestation of HCM, has various causes including ventricular or atrial arrhythmias with fast ventricular response, conduction abnormalities, LVOT obstruction, myocardial ischemia, abnormal vascular reflexes, and hypovolemia. Syncope during/after exertion is suggestive of a cardiac cause, as opposed to syncope after a large meal or prolonged standing, the latter being more suggestive of a reflex-mediated mechanism [6].

Palpitations, often encountered in HCM patients, are the expression of supraventricular or ventricular arrhythmias. The most frequent supraventricular arrhythmias include atrial fibrillation, supraventricular ectopic beats, and other supraventricular-sustained tachycardias. LA enlargement, atrial fibrosis, and diastolic dysfunction have been incriminated as responsible for these occurrences [57]. Ventricular arrhythmias are also frequent and can be life-threatening, especially considering the fact that the underlying mechanism is largely unknown (possible ventricular remodeling secondary to hypertrophy, myocyte disarray, myocardial ischemia, and/or interstitial fibrosis). Ventricular tachycardia can degenerate into ventricular fibrillation, the usual cause of SCD in these patients [1,6].

## 5. Modifiers of Clinical Expression

Given the high variability in terms of clinical expression and illness progression in HCM patients harboring identical causal mutations, sustained efforts have been made to pinpoint factors that act as disease modifiers.

At the present time, it is widely acknowledged that the HCM phenotype is the result of multifaceted interactions between genetic backgrounds, demographical characteristics, environmental influences, and associated comorbidities. Besides, there is a special place to be kept for epigenetics, covering all the modifications that regulate gene expression without altering the genomic sequence, including DNA methylation, histone modification, noncoding RNAs (ncRNAs), or post-translational modifications. It is worth to mention that ncRNAs are regulators of gene expression, but they are also emerging biomarkers of disease progression and potential therapeutic targets. For logical reasons, the first two roles will be discussed together in this paper.

### 5.1. Genetic Modifiers

As opposed to mutations in causative genes, allelic variation in “modifier genes” has been proven to influence the expression of the disease while having little or no effect in normal subjects. Noteworthy, modifier gene polymorphisms are partially responsible for the interindividual variability in subjects sharing identical causal mutations [51].

Among the most studied modifiers of the HCM phenotype are the components of the renin-angiotensin-aldosterone system (RAAS), such as angiotensin-converting enzyme (ACE), angiotensinogen (AGT), angiotensin II receptor type 1 (AGTR1), and aldosterone [58].

Many studies have been performed, with results being difficult to interpret considering the high heterogeneity in terms of study design, environmental background, and causative mutations. Existing evidence suggests that the impact of RAAS genotypes on the HCM phenotype might be dependent on the disease gene. For example, in a cohort of 389 unrelated HCM patients tested for mutations in 8 causal genes, no clinical parameter was found to be associated with the *ACE* genotype. When subset analyses have been performed, significant positive correlations of the homozygous deletion of ACE (DD-*ACE*) genotype have been identified only in patients with mutations in the *MYBPC3* gene [59]. To shed light on the link between *ACE* polymorphisms and HCM, Yuan and colleagues conducted a meta-analysis of 15 studies, counting 2972 genotyped subjects (1047 cases and 1925 controls) [60]. Their findings indicated patients of Asian and Caucasian ethnicity sharing the DD-*ACE* genotype had a higher risk of HCM.

In addition to RAAS, other plausible modifiers of HCM expression have been proposed: ankyrin-2 (ANK2) [36], integrin alpha 8 (ITGA8), ankyrin repeat protein (CARP) [61], voltage-dependent l-type calcium channel subunit beta-2 (CACNB2) [62], ryanodine receptor 2 (RYR2) [63], sodium channel α-subunit gene (*SCN5A*) [63], Muscle ring-finger protein-1 (MURF1), and Muscle ring-finger protein-2 (MURF2) [64]. For instance, it has been shown that *ANK2* rare variants were associated with severe LVH, while patients harboring *SCN5A* rare variants were more likely to have LVOT obstruction and LA enlargement [36].

### 5.2. Other Modifiers

In healthy subjects harboring pathogenic genetic variants, phenotypic expression is likely to emerge only after interplaying with specific factors that modify the relationship with risk [65].

Age is probably the best documented factor in this respect. Large cohort studies showed that most patients developed electrocardiography (ECG) and echocardiographic signs after puberty, with an average age at diagnosis varying between the beginning of the fourth and fifth decade of life [66,67,68,69,70].

Gender is another factor studied; it is to emphasize that although men were more prevalent in published cohorts [66,67,68,69,70,71,72], women generally had worse prognosis. Population-based studies revealed not only that women were diagnosed at older age, had more obstructive physiology, more symptoms, and worse cardiopulmonary exercise tolerance [72,73,74,75], but they also had reduced survival [72,73,74]. These data advocate that more intense diagnostic and therapeutic strategies are needed in HCM women. One of the reasons why the malady is diagnosed at a more advanced age in women could be related to the protective effect exerted by estrogen on myocardial hypertrophy as opposed to androgen, which is pro-hypertrophic [65].

The role of exercise in disease development is still a subject of debate. Different findings might be explained—at least in part—by different intensity levels of physical activity. In animal models, voluntary exercise prevented fibrosis, myocyte disarray, and induction of markers of hypertrophy when started before an established HCM phenotype [76], while long-term intensive exercise training was associated with cardiac fibrosis, changes in ventricular function, and increased arrhythmia inducibility [77]. Although none of these effects has yet been confirmed in humans, based on evidence showing that HCM is the most common cause of SCD in young athletes [78,79], current consensus statements recommend against participating in competitive sports as well as engaging in intense physical activities [6,80,81]. Conclusive answers are expected to be offered by ongoing studies (such as LIVE-HCM, ClinicalTrials.gov Identifier: NCT02549664) assessing the impact of exercise on the well-being of individuals with HCM.

Among the most discussed comorbidities that could influence the natural history of HCM are arterial hypertension and obstructive sleep apnea (OSA), respectively. Apart from difficulties generally aroused in differentiating hypertensive heart disease from HCM [82], the presence of hypertension may negatively affect condition progression by promoting additional LVH through various mechanisms, such as increased afterload and neuroendocrine activation [65,83].

As for HCM and OSA, it has been indicated to be a particularly common and harmful combination. In a review by Nerbass and coworkers, OSA has been reported in 32% to 71% of patients with HCM; moreover, the patients suffering from both HCM and OSA had worse structural and functional impairment of the heart, increased prevalence of atrial fibrillation, and worse quality of life [84]. These findings are not surprising, considering that the two aforesaid maladies share some of the underlying key pathophysiological mechanisms, including overstimulation of the sympathetic nervous system, myocardial hypertrophy, and LA dilation. Increased activation of the mentioned pathways explains the deleterious effects of OSA on HCM pathophysiology, resulting in deteriorated hemodynamics and augmented arrhythmogenesis [65].

### 5.3. Epigenetics

#### 5.3.1. DNA Methylation/Demethylation

An important mechanism in the regulation of gene expression is DNA methylation. Increased methylation of CpG islands of *MYBPC3* drives genetic instability and, thus, deamination of this region leading to mutation, from a cytosine to a thymine [85]. Directly linked to the mentioned mutation, a treatment proposed demethylation with agents such as 5-azacytidine, showing favorable effects such as antifibrosis and anti-hypertrophy [86], of course with all the adverse effects of this drug.

#### 5.3.2. Histone Modification

By interacting with DNA, histones are the foremost players in maintaining chromatin in a silenced or active state. Although various biological factors involved in histone modification have been studied in relation with cardiac hypertrophy [87,88,89,90], only scarce data are available regarding their role in HCM. For instance, it has been shown that brahma-related gene-1 (*Brg1*), a chromatin-remodeling protein that interacts with histone deacetylases and poly(ADP-ribose) polymerase-1, is positively correlated with the severity of HCM and changes in myosin heavy chain expression [91].

Furthermore, demethylation by the switch on of Jumonji domain 2 (JMJD2A)—a lysine trimethyl-specific histone demethylase—induces hypertrophy, and inactivation lessens the phenotype of hypertrophic cardiomyopathy on transverse transaortic constriction-induced models. This enzyme demethylates the trimethylated lysine 9 (H3K9m3) and lysine 36 (H3K36m3) of histone H3, inducing hypertrophy and heart failure [92].

#### 5.3.3. Noncoding RNAs

A growing body of evidence endorses ncRNAs as key regulators of heart pathophysiology. Because of the variable penetrance and high clinical heterogeneity, the levels of lncRNA and miRNAs are constantly changing depending on the stage of the disease: asymptomatic, mild asymptomatic hypertrophy, or defined HCM.

MiRNAs are small, noncoding RNAs of about 22 nucleotides, which negatively regulate gene expression by silencing the complementary mRNA [93]. Bearing in mind the histopathological hallmarks of HCM, it is no wonder that myocardial fibrosis and hypertrophy-related miRNAs have been the most studied in both HCM animal models and patients, revealing increased levels of profibrotic and pro-hypertrophic miRNAs as well as decreased miRNAs inducing opposite effects. Efforts have been made to identify single or panels of miRNAs that could speed up diagnostic work-up.

In the early, symptom-free phases, the miRNAs profile is difficult to evaluate in human hearts, and no such clinical studies have been conducted until now, so the only available information has been provided by studies assessing miRNAs in animal hearts. In a mouse model of HCM, time-course analysis indicated downregulation of miR-1 and miR-133 starting with the pre-disease stage and prior to the upregulation of target genes, with reduced levels sustained throughout disease development [94]. MiR-1 and miR-133 exert protective anti-hypertrophic effects by targeting multiple hypertrophic signaling molecules, such as heart-type fatty-acid-binding protein (FABP3), transforming growth factor beta (TGFβ), and angiotensin II receptor type 1 (AGTR1) [95]. Regardless of how appealing it would be to routinely employ such miRNAs for a timely diagnosis of at-risk subjects, this will only be feasible if the required tissue could be harvested in a minimally invasive manner. Decreased concentrations of miR-1 and miR-133 were reported also in affected heart tissues of patients with defined HCM [96]. Noteworthy, when assessed in plasma, both the aforesaid miRNAs proved to have an increased concentration, most likely as result of cardiomyocyte death during ischemic episodes [97]. Moreover, it has been hypothesized that circulating miR-1 and miR-133 could also be augmented in asymptomatic patients, but further studies are warranted for confirmation.

As for defined HCM, several miRNAs signatures have been described in tissues obtained during septal myectomy. Existing data advocate increased concentrations of miR-21 [98], miR-130b [98], miR-132 [98], miR-222 [99], and miR-96 [99], while miR-1, miR-10b [99], miR-133b [96], miR-139-5p, miR-150, and miR-451 [98] are downregulated.

Although miRNAs within heart tissue are of great scientific interest, the circulating ones are of greater clinical value due to accessibility through minimal-invasive techniques, high stability, and long half-life within the sample as well as fast and accurate detection [100]. Hence, sustained efforts are being made to identify and validate circulating miRNAs with high specificity and sensitivity that are able to facilitate early diagnosis and discriminate pathologies with similar phenotypes.

Among 21 miRNAs examined in plasma samples collected from HCM patients and age- and sex-matched healthy controls, only miR-29a was shown to be related both with hypertrophy and fibrosis, while other miRNAs (i.e., miR-199a-5p and miR27a) were related only with hypertrophy. Notably, when measured in patients with severe symptomatic aortic stenosis, plasma concentration of miR-29a was not augmented compared to the control group, suggesting that the correlation might be disease-specific [101]. Furthermore, Derda and colleagues reported that a specific signature of miRNAs helped distinguish between aortic stenosis, senile cardiac amyloidosis, and obstructive and nonobstructive hypertrophic cardiomyopathy. They identified a significant increase in miR-29a levels only in subjects with obstructive HCM, with robust correlation between miR-29a and the size of interventricular septum [102].

Circulating microRNAs are potential markers of fibrosis, especially in combined panels, as proved in a study by Fang and colleagues. The authors assessed the association between various circulating miRNAs and diffuse fibrosis quantified by cardiac magnetic resonance (CMR) in HCM patients. They discovered that 14 miRNAs, including miR-29a-3p, were upregulated in diffuse fibrosis. The diagnostic value of each miRNA taken separately was only moderate, but when eight miRNAs were considered together, the diagnostic value was substantially improved [103].

Another type of ncRNAs are lncRNAs—RNA transcripts longer than 200 nucleotides that have no protein coding function but are able to influence gene expression at transcriptional and post-transcriptional levels. In recent years, various lncRNAs have been proven to be implicated in HCM development through the control of chromatin remodeling and competitively combining with the corresponding miRNAs, with most studies being conducted in preclinical settings.

In a mouse model of cardiac hypertrophy, terminal differentiation-induced noncoding RNA (TINCR) was found to attenuate LVH by silencing Ca^2+^/calmodulin-dependent protein kinase II (CaMKII), a Ca^2+^/calmodulin-dependent protein kinase mainly expressed in the heart [104], which is recognized as an inducer of cardiac hypertrophy [105,106]. Furthermore, a lncRNA transcript from the *MYH7* locus, called Myheart, has been disclosed to regulate the balance between α and β-MHC expression, preventing pathological LVH [91,107].

As for human evidence, in a very recent paper, Zhou and colleagues identified an inverse correlation between the levels of lncRNA myocardial infarction associated transcript (MIAT) and miR-29a expression in HCM patients with or without myocardial fibrosis [108]. The investigators reported significantly higher expression of lncRNA-MIAT and reduced level of miR-29a in fibrosis (−) subjects compared to fibrosis (+) ones. These statistics are in line with previously reported data showing that lncRNA-MIAT may regulate the expression of miR-29a-3p by acting as an endogenous miRNA sponge [109].

Another study (including seven HCM patients and five healthy controls) confirmed that lncRNA-related impairment of translational regulation could be one of the mechanisms involved in HCM pathogenesis [110]. Among the 8435 lncRNAs detected in heart tissue samples, 965 lncRNAs were upregulated and 461 lncRNAs were downregulated in HCM subjects. Importantly, lncRNA-coexpressed mRNAs were mostly enriched in ribosome- and oxidative phosphorylation-associated processes, resulting in disrupted expression of numerous proteins.

### 5.4. Post-Translational Regulation

Post-translational modifications represent an important mechanism by which a variety of cellular processes and systems are regulated. For example, phosphorylation of the regulatory light chain (RLC) of cardiac myosin by myosin light chain kinase facilitates actomyosin interaction and increases force at the level of the muscle. HCM caused by mutations in the region A13T of RLC (the site close to Ser15 where phosphorylation takes place) induces abnormal calcium-binding of RLC and reduces the alpha-helix content (a modification well compensated by phosphorylation), highlighting that phosphorylation contributes to compensate some pathological consequences of HCM mutations [111]. The extent of RLC phosphorylation can be a diagnostic method, as in cases with LVH and normal LVEF, RLC phosphorylation is normal, compared with dilated left ventricles and reduced LVEF where RLC phosphorylation is significantly reduced [112]. Therefore, increasing RLC phosphorylation seems to protect the heart by increasing the contractility aiming to adapt it to stress [113].

In addition to phosphorylation, other post-translational modifications of sarcomeric proteins, such as S-glutathionylation, acetylation, or citrullination, can contribute to the pathophysiology of HCM.

Acetylation of certain sites within a 40 kDA fragment of cMyBP-C affects contractility and the function of the actomyosin complex [114]. S-glutathionylation of cMyBP-C in the heart tissue of a hypertensive mouse model was correlated with the diastolic dysfunction developed in these animals [115].

## 6. Modalities to Detect Clinical Expression

### 6.1. Echocardiography

Echocardiography has traditionally played a main role in the diagnosis and prognostic assessment of this complex disease as well as in formulation of various management strategies. Following clinical exam and ECG studies, echocardiography is the third step in the diagnostic workup of a patient with suspected HCM by confirming the presence of LVH.

**Figure 2:**
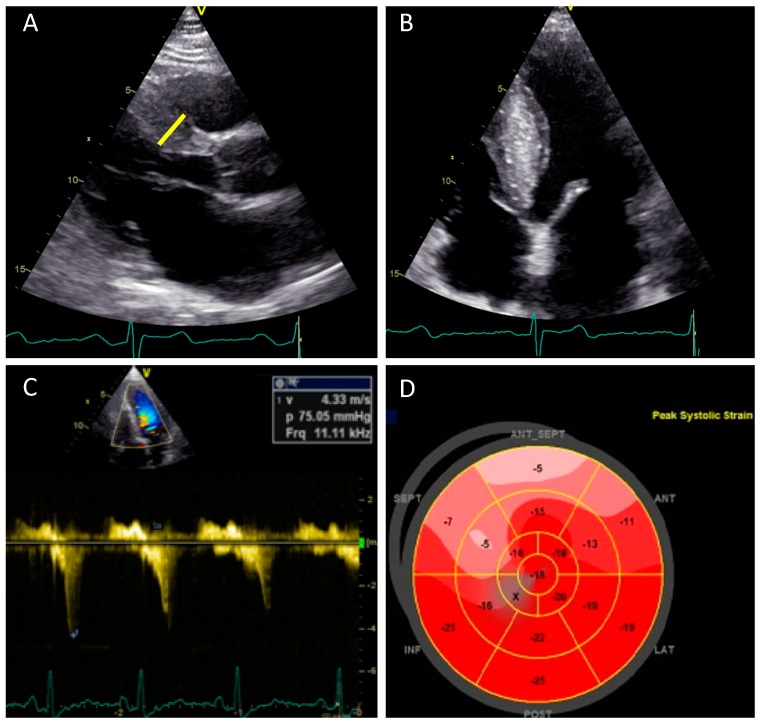
The role of transthoracic, two-dimensional echocardiography in the assessment of hypertrophic cardiomyopathy (HCM). (**A**) A parasternal long axis view showing basal septal hypertrophy (maximum LV wall thickness 21 mm—yellow line). (**B**) Apical four-chamber view shows hypertrophy of the interventricular septum. (**C**) Continuous wave Doppler from modified apical five-chamber view showing a rest dynamic gradient of 75 mmHg in the LVOT (**D**) A bull’s eye plot of two-dimensional speckle tracking showing reduced peak longitudinal strain values in the septum—the areas most affected by hypertrophy with preservation of the basal-to-apical gradient. HCM—hypertrophic cardiomyopathy, LV—left ventricle, and LVOT—left ventricular outflow obstruction.

Diagnosis of HCM can be made in the majority of patients with two-dimensional transthoracic echocardiography (TTE) when an increased LV wall thickness >15 mm is detected with a nondilated cavity and in the absence of any other condition known to cause LVH of that magnitude (systemic hypertension or aortic stenosis) [6,116] (Figure 2A,B). In relatives of patients with HCM, documenting mild increases in LV wall thickness (12–14 mm) can be considered diagnostic. Increased right ventricle free wall thickness (>8 mm) is present in over one-third of patients with HCM [117]. Additional diagnostic criteria have been defined as septal to a posterior wall thickness ratio >1.3 in patients with normal blood pressure or septal to a posterior wall thickness ratio >1.5 in hypertensive patients [6,116]. Nevertheless, genotype-positive adults, including those who suddenly die, may have normal or near normal wall thickness [42]. It should be emphasized that asymmetrical LV hypertrophy is not pathognomonic of sarcomeric HCM, but it may be encountered in a variety of other congenital or acquired conditions like systemic hypertension, pulmonary hypertension, aortic stenosis, septal sarcomas, amyloidosis, Fabry disease, Friedrich ataxia, mucopolysaccharides, or glycogen storage disorders [118].

The most common locations for increased wall thickness in HCM are the basal anterior septum, the anterior free wall, and the posterior septum at the midlevel. Although LVH involves most of the myocardium in the majority of patients, a minority (10%) of HCM subjects exhibit increased LV thickness confined to only one or two LV segments. Although, typically asymmetric in distribution, any pattern of LV thickening can be seen in HCM from apical LVH to concentric LVH (1%).

Echocardiography also asserts the presence of SAM of the anterior mitral leaflet itself, or of the attached chordae, with or without developing a gradient across the LVOT. Although not pathognomonic, this is highly indicative of HCM, with a specificity approaching 99% [119]. It may be also encountered in other congenital or acquired circumstances like transposition of great vessels, hypercontractile conditions, after mitral valve repair, antero-apical infarction with akinetic or hypokinetic apical segments and compensatory hyperkinesis of the basal segments, and in elderly women with hypertension or sigmoid septum under hyperdynamic conditions [120]. Originally, SAM was put on behalf of the “Venturi” effect, but now it has been demonstrated that the hyperdynamic forces of flow are rather the dominant, implicated mechanism [121].

Closely related with SAM is mitral regurgitation (MR), which is usually functional in HCM and occurs as a result of SAM of the anterior leaflet, even if there are interindividual differences in the degree of MR for comparable degrees of SAM [122]. This variability occurs as a result of the mobility and length of the posterior mitral valve; a limited posterior leaflet excursion is associated with a more severe mitral regurgitation for the same degrees of SAM [123]. In most cases, MR due to SAM is directed posteriorly and is rarely severe. Another direction of the jet or a severe regurgitation should raise the suspicion of another associated mechanism, and transesophageal echocardiography (TEE) should be used along with TTE for clarifying the mechanism. In this case, intrinsic valvular abnormalities should be searched for such as: leaflet fibrosis, leaflet restriction secondary to “percussion injury” [124], a chordal rupture, or an associated myxomatous disease [125]. LVOT obstruction assessment at rest or during provocation results in a characteristic signal with a “dagger” shaped appearance showing late peaking flow in LVOT [126] (Figure 2C). Subaortic (i.e., LVOT) obstruction in HCM is due to SAM of the mitral valve contacting the ventricular septum in mid-systole. This creates mechanical impedance to blood flow as it exits the heart, resulting in a pressure gradient between the LV cavity and the aorta. Morphological features that contribute to the development of LVOT gradients are: narrowing of the outflow tract due to excessive thickening of the interventricular septum, apical displacement of papillary muscles, and elongated anterior leaflet of the mitral valve.

It is clinically important to distinguish between the obstructive or nonobstructive forms of HCM because management strategies depend highly on this issue. Resting LVOT obstruction, defined as peak gradient >30 mmHg, is an important predictor of death and heart failure progression [54]. Up to one-third of patients with HCM will have peak gradients >30 mmHg on rest, another third will have labile, physiologically provoked gradients (<30 mmHg at rest and >30 mmHg with physiologic provocation), and the final third will have the nonobstructive form of HCM [127]. Marked gradients >50 mmHg at rest or with provocation represent the threshold for percutaneous or surgical intervention if symptoms cannot be controlled with medications [6,116]. Exercise (stress) echocardiography using a standard symptom-limited Bruce protocol is the preferred method for provoking LVOT gradient, as this mimics most closely the conditions patients experience on a daily basis. Additional echocardiographic features that can be encountered in LVOT obstruction are mid-systolic notching, early systolic closure of the aortic valve, coarse fluttering of the aortic valve during systole, and fibrotic mitral valve changes due to contact of the leaflets with the septum [126].

In addition to the classical variant of HCM with or without LVOT obstruction, a morphological variety of other HCM exists, some of which may be managed differently: midcavity obstruction, midcavity obstruction with apical aneurism, apical HCM, or right ventricular obstruction.

Midcavity or midventricular obstruction with or without apical aneurism results from different anatomic and hemodynamic elements [128,129]. Midcavity obstruction in HCM occurs because of the apposition of the septum and lateral wall or because of the systolic apposition of hypertrophied papillary muscle and lateral wall at the level of the mid-LV, producing two distinct LV chambers (i.e., proximal and distal with an “hour glass” shape LV), and associated with an LV apical aneurysm of varying size [130]. Echocardiography in isolated midcavity HCM shows absence of systolic anterior motion of the mitral valve and a narrowed mid-LV cavity with or without a thin-walled, scarred LV apical aneurysm, sometimes containing thrombi.

Apical HCM is another uncommon morphologic variant of HCM in which hypertrophy of the myocardium predominantly involves the apex of the LV [131]. This form can be missed by echocardiographic evaluation due to foreshortening of the apex. CMR is most accurate in diagnosing this distinct morphological type of HCM.

A very small number of HCM patients demonstrate obstruction to flow in the right ventricular (RV) outflow tract. This is usually a result of midsystolic contact of prominent right ventricular muscle bundles located in the RV outflow tract region.

Another important parameter to be measured by echocardiography is LA, as retrospective and observational echocardiographic studies have demonstrated that increased LA size is associated with adverse events with marked LA enlargement >48 mm (transverse linear dimensions) or volume >118 mL being correlated with higher risk for heart failure, death, or atrial fibrillation [132].

LA volume depends on the presence of diastolic dysfunction, mitral regurgitation, and on the coexistence of atrial myopathy. LA diameter is included in the risk stratification score developed by the European Society of Cardiology [6], although the independent relationship between LA size and sudden death risk in HCM is unresolved and cannot be used as a unique measurement for this purpose [116].

The assessment of ventricular diastolic and systolic function plays an essential role in the prognostic stratification and management of patients.

A reduction of chamber compliance and increased stiffness associated with reduced LV volume due to excessive hypertrophy are the main pathophysiological mechanisms in patients with HCM. Doppler echocardiography of the LV inflow tract allows the characterization of diastolic function in HCM, most frequently indicating impaired LV relaxation. Nishimura et al. showed that classic Doppler evaluation does not provide accurate information about filling pressures in HCM [133]. On the other hand, additional diastolic-derived parameters, such as the ratio of E to E’, color M mode flow propagation velocity, and peak E wave-to-flow propagation velocity ratio, were shown to predict LV end-diastolic pressure measured invasively during catheterization, as opposed to pulmonary venous velocities or atrial volumes which did not [134]. These parameters also seem to reliably predict exercise tolerance [135] and reduction in filling pressures after septal ablation or myomectomy [136].

In the majority of HCM patients, LV systolic function is normal or supernormal in terms of LVEF. The emergence of novel echocardiographic techniques, such as myocardial strain, provides an opportunity to assess intrinsic regional and global myocardial mechanics and function [137]. In addition, abnormalities in myocardial mechanics precede the development of clinical hypertrophy in patients with the presence of sarcomeric mutations [138]. With the use of strain imaging, it is now possible to identify regional heterogeneity in contractile functions in order to better understand the myocardial mechanics in HCM.

Significant impairment of longitudinal contractile function despite normal LVEF was demonstrated in a number of studies that showed abnormal reduced global longitudinal strain (GLS) values in HCM, but with preservation of basal to apical gradient (Figure 2D), an increase in circumferential strain, normal systolic twist or torsion, and reduction of untwisting in diastole [139]. In contrast, Reddy at al. reported systolic “paradoxical” lengthening in the longitudinal strain of the apical segments and attenuation of longitudinal strain values in the mid and distal segments of LV, findings that suggest a loss of base-to-apex gradient in the apical HCM form [126,140]. In one population of patients with nonobstructive HCM and no evidence of LVOT obstruction at baseline, higher GLS values (i.e., less negative) were associated with a greater likelihood of new or progressive heart failure [141]. However, the precise role of GLS in large populations of patients with HCM remains to be clarified.

An increasingly important subgroup of patients (10%) with nonobstructive HCM in tertiary centers will develop the end-stage phase of HCM defined by LVEF <50% [142]. This stage of disease is characterized by myocardial thinning, systolic impairment, and LV cavity dilation, and it is related to myocardial scarring, being associated with increased mortality and sudden cardiac death [143].

Tissue Doppler imaging (TDI) has become standard in the echocardiographic evaluation of patients with HCM. Systolic myocardial velocity has been shown to be reduced in HCM, even in non-hypertrophied segments [144]. In addition, early diastolic mitral annular velocities are lower in patients with HCM, compared with age-matched controls, and relate to LV hypertrophy [135]. Regarding patients with sarcomeric mutations and absence of LVH on echocardiographic exam, a systolic TDI velocity in the lateral annulus <13 cm/s is predictive for manifesting HCM with a sensitivity of 100% and a specificity of 93% [145]. In addition, the LVOT peak gradient is negatively correlated with systolic and early diastolic mitral annular velocities, stressing the impact of LVOT obstruction on LV function [146].

### 6.2. Cardiovascular Magnetic Resonance Imaging

Because of its high spatial resolution, irrespective of the body habitus, and its tissue characterization capabilities, cardiovascular magnetic resonance (CMR) has emerged as a valuable tool for the assessment of patients with HCM and their relatives [147]. Noncontrast CMR is able to detect structural findings such as myocardial crypts or apical-to-basal muscle bands, which may not be visible with echocardiography but are currently considered features of genotype-positive pre-hypertrophic HCM [148,149,150]. Moreover, contrast CMR can accurately detect myocardial fibrosis, which is the hallmark of HCM, linked to adverse outcomes such as sudden death, ventricular arrhythmias, or heart failure.

One of the most important clinical applications of CMR is the differentiation of HCM from other etiologies of LVH such as hypertensive heart disease, cardiac amyloidosis, or Anderson–Fabry disease. CMR may be especially helpful in differentiating HCM from athlete’s heart; in HCM the extracellular volume (ECV) is increased due to interstitial fibrosis, whilst in athlete’s heart, the ECV is decreased due to cellular hypertrophy [151].

Recently, it was demonstrated that there are no phenotypic differences between *MYH7*- and *MYBPC3*-associated hypertrophic cardiomyopathy when assessed by CMR imaging [152]. LV volumes, mass, maximal wall thickness, morphology, LA volume, and mitral valve leaflet lengths as well as the presence and the proportion of late gadolinium enhancement (LGE) were similar in both genetic patients [152].

**Figure 3:**
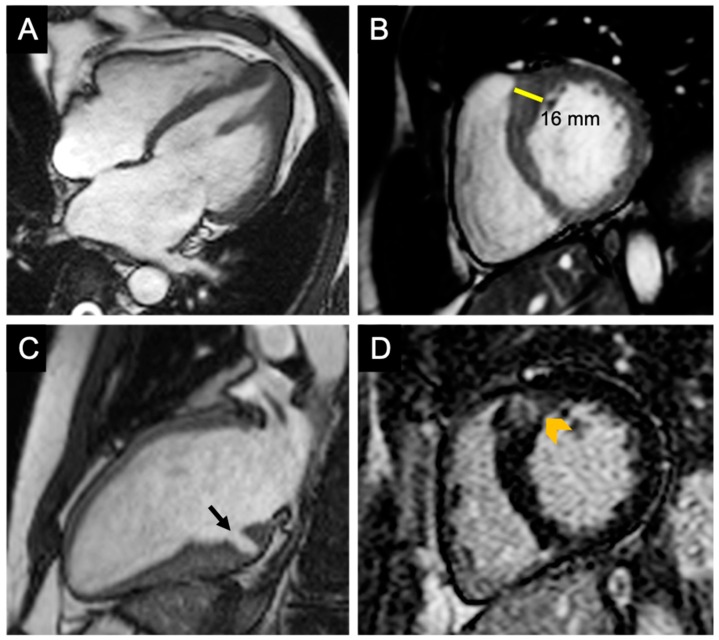
The role of CMR in the assessment of HCM. (**A**) Cine-CMR four-chamber view—diastolic phase demonstrating hypertrophy of the apical segments of the left ventricle and apical insertion of the papillary muscle in a patient with apical HCM. (**B**) Cine-CMR short-axis view—diastolic phase demonstrating hypertrophy of the confluence of the basal anterior septum with the contiguous anterior free wall; this is the most common location of LV hypertrophy in patients with HCM. (**C**) Cine-CMR apical two-chamber view—diastolic phase showing a myocardial crypt (black arrow) located in the basal inferior wall in a patient without definite criteria for HCM (the maximum wall thickness was 13 mm). (**D**) LGE short-axis view of the patient in panel B, demonstrating patchy hyperenhancement of the hypertrophied segment indicating replacement fibrosis. CMR—cardiovascular magnetic resonance, HCM—hypertrophic cardiomyopathy, LV—left ventricle, and LGE—late gadolinium enhancement.

Short-axis cine images are useful for measuring LV wall thickness, volumes, and LVEF. Because of its high contrast between the myocardium and blood pool, CMR permits an accurate measurement of the segments poorly visible with echocardiography such as the basal anterior segment and the apical segments (Figure 3A,B). Most frequently, hypertrophy is localized at the level of the contiguity of the basal antero-septal wall with the basal anterior wall [153] (Figure 3B).

CMR is able to identify myocardial crypts, defined as invaginations that penetrate more than 50% of the compact myocardium at end-diastole and collapse at end-systole [150] (Figure 3C). Usually, they are multiple and located in the basal inferior LV wall. Myocardial crypts may be also encountered in the healthy population; however [149], they are more prevalent in patients with genotype-positive HCM, with and without hypertrophy [150].

Another imaging biomarker that is easily identified with CMR in patients with HCM is the apical-to-basal muscle band. This unique structure was detected in 60% of phenotype-positive HCM patients as well as in genotype-positive, phenotype-negative family members [148].

Anomalies of the mitral valve apparatus may also be assessed by CMR. Apical insertion and other conformational anomalies of papillary muscles, as well as elongation of the anterior mitral valve, may be subtle findings encountered in genotype-positive, even in pre-hypertrophic, stages (Figure 3A).

The addition of gadolinium-based contrast agents (GBCA) permits the detection and quantification of myocardial fibrosis. Two different types of myocardial fibrosis may be encountered in the setting of HCM: replacement fibrosis (scar), which appears to be linked to periods of ischemia and cardiomyocyte necrosis; this type of fibrosis can be detected on LGE sequences (Figure 3D); and interstitial fibrosis—a reactive, diffuse process identifiable by T1 mapping techniques; the measurement of T1 relaxation times before and after GBCA administration is able to estimate the extracellular volume fraction (ECV) [154].

Both types of myocardial fibrosis may appear early in the course of disease, even in pre-hypertrophic stages. Myocardial ECV, as a measure of interstitial fibrosis, is increased in HCM sarcomere mutation carriers even in the absence of LVH, suggesting that fibrosis is an early stage in the natural history of HCM, and it is not necessarily linked to hypertrophy [155]. In keeping with this theory, serum levels of biomarkers of collagen synthesis (C-terminal pro-peptide of type I procollagen (CICP)) were significantly higher in mutation carriers without LVH [40].

On the contrary, LGE is very rarely seen in mutation carriers before the development of LVH [155,156]; however, it is visualized in >60% of patients with clinically overt HCM likely representing replacement fibrosis [155].

Stress perfusion CMR permits the assessment of inducible myocardial ischemia in HCM patients. It was shown that this population of patients had decreased coronary flow reserves as an expression of microvascular disease. Patients with higher degrees of hypertrophy have more severe microcirculatory dysfunction [157].

### 6.3. Biomarkers

One aspect of uppermost importance in HCM is the prevention of SCD. Identification of high-risk biomarkers for ventricular arrhythmias or evolution from stable disease to LV dysfunction and heart failure allows prompt identification of HCM individuals with elevated likelihood of SCD.

Identification of necrosis, fibrosis, or stress biomarkers would assure a better risk stratification. Many biomarkers from different categories such as high-sensitivity cardiac troponin T or I (hs-cTnT, hs-TnI), N-terminal pro-b-type natriuretic peptide (NT-pro-BNP), growth differentiation factor 15 (GDF-15), Galectin-3 (Gal-3), CICP, or soluble suppression of tumorigenicity (soluble ST2) have been investigated.

Necrosis markers such as troponin are very sensitive and specific for myocyte injuries, being indicators of disease progression and worse prognosis [158]. Troponins constantly showed an independent, predictive role of extensive LGE in low- or intermediate-risk HCM cases [159]. Kubo et al. found that troponin T and plasmatic brain natriuretic peptide (BNP), biomarkers of myocyte stress, are complementary biomarkers useful for identifying patients with unfavorable evolution towards heart failure. Other common, accurate stress biomarkers are natriuretic peptides [160]. The plasmatic values of BNP increase in overload conditions or extensive cardiac fibrosis [161]. BNP independently predicts mortality and morbidity in HCM [162], while NT-pro-BNP correlates with the diastolic and systolic dysfunction or LV wall stress [163].

Inflammatory markers such as IL-6 and tumor necrosis factor α (TNF-α) may be implicated in HCM pathogenesis by stimulation of fibrosis pathways. In mice, IL-6 and the IL-6 receptor induces cardiac hypertrophy [164], and probably a similar mechanism is encountered in humans.

Myocardial fibrosis is associated with a higher risk of adverse events, and identification of fibrosis markers would ameliorate the accuracy of risk stratification. Matrix metalloproteinases (MMPs) play an important role in the fibrosis process of remodeling, which is a mechanism already shown after myocardial infarction [165]. The imbalance between MMP and TIMPs (tissue inhibitors of metalloproteinases) could explain the extensive fibrosis seen in advanced HCM. Patients with HCM have high levels of MMP-2, MMP-9, procollagen type III N-terminal pro-peptide (PIIINP), and ICTP indicating an increased turnout of collagen, as well as a reduction of TIMP-1 and increased CICP, showing fibrosis accumulation. On mouse models of hypertensive cardiomyopathy, it was shown that ACE inhibitors impeded fibrosis by targeting MMPs [166], confirmed at least for the diastolic function in nonobstructive HCM [167].

The study of biomarkers gives some insights into the pathophysiology of the disease, but neither their exact function in the clinical assessment of patients nor their potential therapeutic roles are completely understood.

## 7. Perspectives

The discovery of disease modifiers opened new horizons for the treatment of HCM. Malady development could be potentially slowed or even stopped by inhibiting the pro-hypertrophic and profibrotic miRNAs with antimiRs or by stimulating the miRNAs having opposed effects with miRNA mimics. AntimiRs are synthetic oligonucleotides complementary to the mRNA-binding sequence. Different chemical modifications, such as the addition of cholesterol, have been made in order to increase stability and improve the pharmacodynamics or cellular uptake of antimiRs [168]. Some in vivo studies have been conducted where cardiomyocyte hypertrophy has been inhibited by antagomirs for miR-21 [169] and miR-132 [170] on heart failure models induced by pressure overload. Inhibition of miR-34a in mice was shown to reduce atrial enlargement and maintain cardiac function in those with moderate pathology but not in advanced stages [171].

Also, manipulating dysregulated lncRNAs to adjust their expression could improve the course of the disease.

Until ncRNAs may serve as reliable therapeutic targets, there is still a long way to go. Indeed, lncRNAs regulate genetic networks and have no single pathway, so their use is hindered by potential severe, off-target effects. Before entering the clinical arena, robust proof of concept studies in relevant animal models are essential.

## 8. Highlights

The main existing evidence, along with knowledge deficits and proposed strategies to bridge the gaps, are summarized in Table 2.

## 9. Conclusions

Undoubtedly, our knowledge about HCM has significantly improved since its early description in 1958, but there is still a long road ahead until the complete elucidation of underlying physiopathological mechanisms and achievement of individually tailored health interventions.

## Figures and Tables

**Table 1:** Core and emerging genes for hypertrophic cardiomyopathy.

**Core Genes**	**Protein**	**References**
*MYH7*	β-Myosin heavy chain	[8]
*MYBPC3*	Myosin-binding protein C	[8]
*TNNT2*	Cardiac troponin T	[9]
*TNNI3*	Cardiac troponin I	[6]
*TPM1*	α-Tropomyosin	[9]
*ACTC1*	Cardiac muscle α-Actin	[6]
*MYL2*	Myosin light chain 2	[10]
*MYL3*	Myosin light chain 3	[11]
**Emerging Genes**	**Protein**	**References**
*ACTN2*	α-Actin 2	[12]
*ANKRD1*	Ankyrin repeat domain 1	[13]
*CSRP3*	Cysteine and glycine-rich protein 3 or Muscle Lin11/Isl1/Mec3( LIM) protein	[14]
*DES*	Desmin	[15]
*FHL1*	Four and a half LIM domain protein 1	[7]
*LBD3*	LIM domain-binding protein 2	[16]
*MYLK2*	Myosin light chain kinase 2	[17]
*MYO6*	Myosin 6	[18]
*MYOZ2*	Myozenin 2	[19]
*NEXN*	Nexilin	[20]
*MYPN*	Myopalladin	[21,22]
*PDLIM3*	Alpha-Actinin-2-Associated LIM Protein	[22]
*TCAP*	Telethonin	[23]
*TNNC1*	Cardiac troponin C	[24]
*TRIM63*	Tripartite Motif-Containing Protein 63	[15]
*TTN*	Titin	[25]
*VCL*	Vinculin	[26]

**Table 2:** Summary of current knowledge, gaps in evidence, and future directions.

Current Knowledge	Gaps in Evidence	Future Directions
HCM is caused by mutations in sarcomeric or sarcomeric-related genes;	Incomplete understanding of disease genetics, particularly in “genotype negative/phenotype positive” patients;	Use of broad gene panels, or whole-exome/whole-genome sequencing;
Penetrance and expressivity are subjected to various genetic and nongenetic influences;	Incomplete understanding of the natural history of HCM;	Conduct multiethnic, large-scale prospective cohort studies to assess disease progression;
Overt disease can be easily diagnosed with available imaging techniques;	Deficiency/lack of modalities that allow early diagnosis;	Refine existing imaging techniques; develop multimodal approaches that permit early diagnosis;
Noncoding RNAs have emerged as useful tools for diagnosis, prognosis, and therapeutics of HCM;	Limited data about their mechanism of action and regulated pathways, particularly in humans;	Use relevant in vivo models before translating into clinics; Use alternative in vitro models (patient-derived iPSC) to gain insights about regulated pathways in humans;
Risk prediction models are used in clinical practice (HCM Risk-SCD score).	Risk stratification models in specific groups (such as women and children).	Fine-tune existing risk models according to findings from multiethnic, large-scale cohort studies focusing on specific subpopulations.

HCM—hypertrophic cardiomyopathy, RNA—ribonucleic acid, SCD—sudden cardiac disease, and iPSC—induced pluripotent stem cells.
